# Outcome Risk Factors during Respiratory Infections in a Paediatric Ward in Antananarivo, Madagascar 2010–2012

**DOI:** 10.1371/journal.pone.0072839

**Published:** 2013-09-12

**Authors:** Soatiana Rajatonirina, Norosoa Harline Razanajatovo, Elisoa Hariniana Ratsima, Arnaud Orelle, Rila Ratovoson, Zo Zafitsara Andrianirina, Todisoa Andriatahina, Lovasoa Ramparany, Perlinot Herindrainy, Frédérique Randrianirina, Jean-Michel Heraud, Vincent Richard

**Affiliations:** 1 Epidemiologic Unit, Institut Pasteur from Madagascar, Antananarivo, Madagascar; 2 National Influenza Center, Virology Unit, Institut Pasteur from Madagascar, Antananarivo, Madagascar; 3 Centre de Biologie Clinique, Institut Pasteur from Madagascar, Antananarivo, Madagascar; 4 Epidemiologic Unit, Institut Pasteur de Dakar, Dakar, Senegal; 5 Pediatric Ward, Centre Hospitalier de Soavinandriana, Antananarivo, Madagascar; 6 Pediatric Ward, Centre Hospitalier de District de Niveau 2, Moramanga, Madagascar; Alberta Provincial Laboratory for Public Health/University of Alberta, Canada

## Abstract

**Background:**

Acute respiratory infections are a leading cause of infectious disease-related morbidity, hospitalisation and mortality among children worldwide, and particularly in developing countries. In these low-income countries, most patients with acute respiratory infection (ARI), whether it is mild or severe, are still treated empirically.

The aim of the study was to evaluate the risk factors associated with the evolution and outcome of respiratory illnesses in patients aged under 5 years old.

**Materials and Methods:**

We conducted a prospective study in a paediatric ward in Antananarivo from November 2010 to July 2012 including patients under 5 years old suffering from respiratory infections. We collected demographic, socio-economic, clinical and epidemiological data, and samples for laboratory analysis. Deaths, rapid progression to respiratory distress during hospitalisation, and hospitalisation for more than 10 days were considered as severe outcomes. We used multivariate analysis to study the effects of co-infections.

**Results:**

From November 2010 to July 2012, a total of 290 patients were enrolled. Co-infection was found in 192 patients (70%). Co-infections were more frequent in children under 36 months, with a significant difference for the 19–24 month-old group (OR: 8.0).

Sixty-nine percent (230/290) of the patients recovered fully and without any severe outcome during hospitalisation; the outcome was scored as severe for 60 children and nine patients (3%) died.

Risk factors significantly associated with worsening evolution during hospitalisation (severe outcome) were admission at age under 6 months (OR = 5.3), comorbidity (OR = 4.6) and low household income (OR = 4.1).

**Conclusion:**

Co-mordidity, low-income and age under 6 months increase the risk of severe outcome for children infected by numerous respiratory pathogens. These results highlight the need for implementation of targeted public health policy to reduce the contribution of respiratory diseases to childhood morbidity and mortality in low income countries.

## Background

Respiratory infections are a major cause of infectious disease-related morbidity, hospitalisation, and mortality among children under 5 years old worldwide, and particularly in developing countries [1].

These diseases are responsible for 4 million deaths worldwide every year [4]: they are thus among the leading causes of death and the most common infectious cause of death [2]. Although many episodes of respiratory infection are mild, 7 to 13 percent are severe enough to warrant hospitalisation [3]. These diseases are responsible for at least 6% of the world's disability and death [1]. It is estimate that there are 33.7 million cases of respiratory infection annually among African children and longitudinal studies suggest that the overall mortality rate among children in developing countries is between 6.6% and 14.1% [5].

Despite respiratory infections being a public health priority, data on their epidemiology in Africa are sparse. Diagnostic methods to identify the pathogen rapidly are not available in low-incomes settings, so most patient with respiratory infections are treated empirically with antibiotics based on local epidemiology [6]. Administering appropriate therapy at the appropriate time would improve outcomes. These diseases also have an economic impact, as they lead to substantial consumption of antimicrobial agents in both community and hospital settings. Furthermore, the high frequency of respiratory infections and the excessive use of antimicrobial agents are major contributors to the development of antibiotic-resistant pathogens.

Knowing the local epidemiology of respiratory infections is essential if appropriate empiric therapy is to be prescribed. Evaluation of mixed infections and the relative importance of each potential pathogen may also contribute to improving the understanding of the pathogenesis of these infections [7], [8]. Our study aimed to evaluate the risk factors associated with the evolution and outcome of respiratory illnesses in patients aged under 5 years old hospitalised in one of the four main public hospitals in Antananarivo.

## Materials and Methods

### Study site

The study was conducted at the “Centre Hospitalier de Soavinandriana” (CENHOSOA) in Antananarivo, the capital city of Madagascar, which is located in the central highlands. Antananarivo consists of administrative, commercial, industrial and residential areas, with patches of agricultural land, mostly rice fields. The city is divided into six administrative districts. In Madagascar, Hib vaccine was introduced in 2008, and Pneumococcia vaccine in 2012

CENHOSOA is a national referral hospital in the third district of the urban municipality of Antananarivo; hospitalization and care at this hospital is paid for by the patients. It is located near the Institut Pasteur of Madagascar (IPM). The paediatric and neonatology ward of CENHOSOA has 54 beds, and the annual number of children hospitalized for respiratory infection was estimated to be 150.

### Study design

A prospective, hospital-based, study was conducted at CENHOSOA from November 2010 to July 2012.

**Figure 1:**
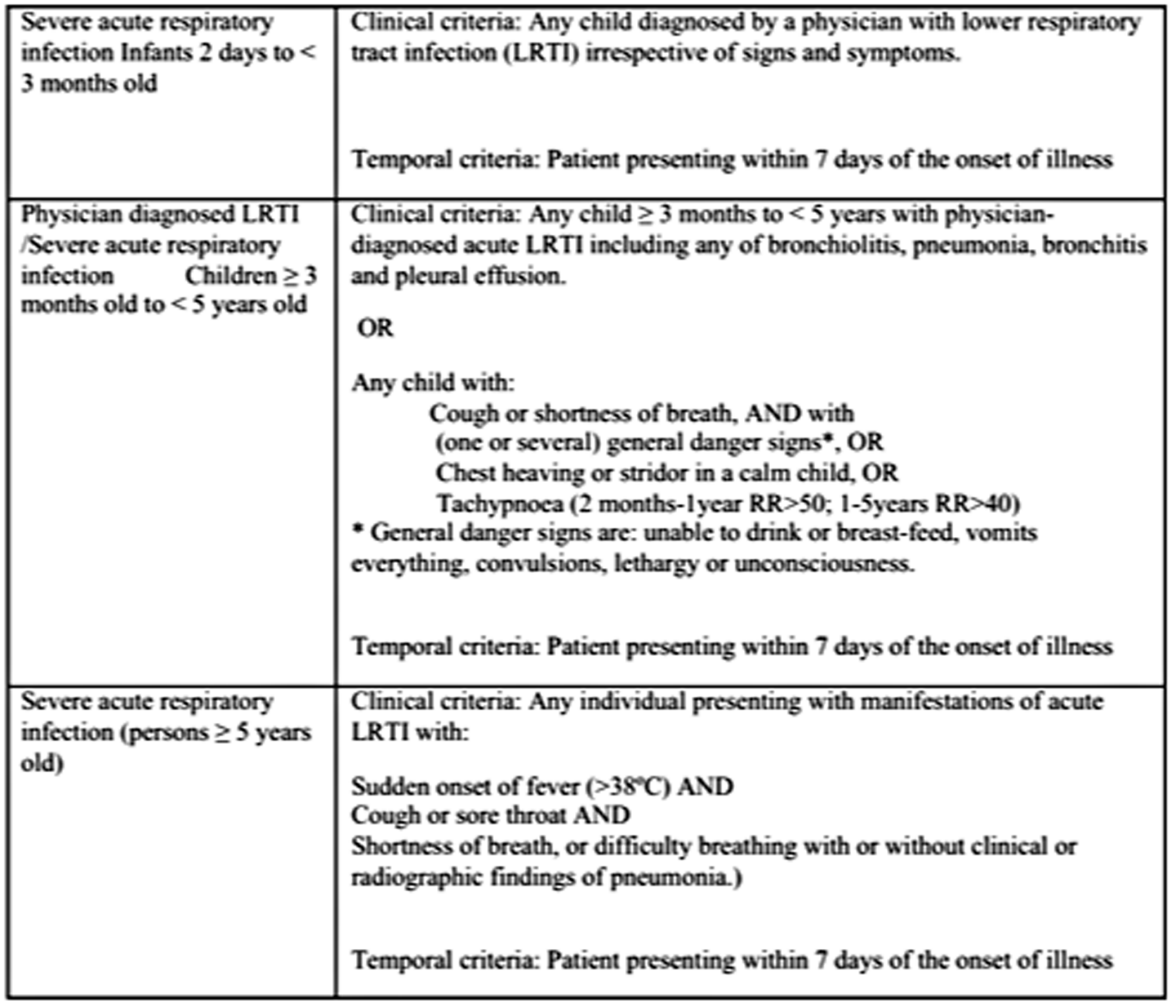
Eligibility criteria for patients included in the study.

The case definition and eligibility criteria used during this study are described in figure 1.

Demographic, socio-economic, clinical and epidemiological data were recorded in case report forms (CRFs). Paediatricians completed the CRFs which were then validated by a clinical research officer. Instruction sheets were provided to clinical research officers to ensure accurate data collection. The data collected was managed by a senior medical epidemiologist.

On admission, trained personnel using standard operating procedures had collected nasal and throat swabs, and sputum sample by aspirating airway secretion from the hypopharynx through the nose or tracheal aspirates. Nasopharyngeal secretions, tracheal aspirates and sputum were obtained before start of antibiotic therapy. Specimens were transported within 30 minutes of sampling to the IPM for tests of respiratory pathogens.

HIV serostatus was not determined; the prevalence of HIV in Madagascar is low, estimated to be around 1 per 1000 [7].

### Ethics statement

The study was approved by the national ethics committee of the Ministry of Health of Madagascar. Written informed consent was obtained from parents or legal guardians of children enrolled in the study. Consenting to be enrolled in the study required consent to provide samples for diagnosis of influenza and other respiratory pathogens and to fill out a case report forms (CRFs). Refused consent and hospitalization within the two weeks before consultation were reasons for exclusion from the study.

### Biological analysis

Virological analyses were performed by National Influenza Centre (NIC).

A panel of multiplex real-time RT-PCR was used for the detection of viral pathogens in nasopharyngeal swabs. Primers and probes were obtained from the authors of reported studies or were developed at the NIC [8]. Viral RNA and DNA were extracted from 140 µl of each specimen using the QIAamp viral RNA mini kit and QIAampMinElute virus kit (QIAgen, Courtaboeuf, France) according to the manufacturer's protocols. An ABI 7500 Fast real-time PCR system (Applied Biosystems) was used for amplifications. Each sample was subjected to five different amplification reactions, each containing primers and probes specific for two or three viruses such that the following 12 respiratory viruses were targeted as previously described: parainfluenza virus 1–3 (PIV), human coronaviruses (hCoV) -OC43, -229E, -NL63 and HKU1, respiratory syncytial virus (RSV), human metapneumovirus (hMPV), human rhinovirus (hRV), adenovirus (AdV) and bocavirus (BoV).

As part of our routine influenza surveillance, screening for influenza viruses is performed by MDCK cell inoculation and real-time RT-PCR according to the CDC protocols [9].

### Bacteriological analyses were performed by the medical analysis laboratory of IPM

Gram-stained sputum preparations were examined for polynuclear neutrophils (PN) and epithelial cells. The areas of maximal purulence were examined and graded as follows: class 1: more than 25 cells and fewer than 10 PN, class 2: more than 25 cells and 10–25 PN, class 3: more than 25 cells and more than 25 PN, class 4: 10–25 cells and more than 25 PN and class 5: fewer than 10 cells and more than 25 PN. Only the classes 4 and 5 present a high positive predictive value. If the laboratory determined that the sample was of oral origin it had been rejected. So, classes 1 to 3 had been considered as rejection criteria.

Quantitative sputum cultures were performed on each specimen by a previously described method [10]: sputum specimens were homogenized with an equal volume of Digest-EUR® on a vortex mixer; 10 µl aliquots of the homogenate were serially diluted in saline and 10 µl aliquots of the dilutions were plated with a sterile inoculator on Blood Columbia agar with nalidixic acid and colistin (ANC), Chocolate Polyvitex (PVX) agar, and Drigalski agar. Plates were incubated under 5% CO_2_ at 35°C for 24 to 48 hours. Colonies growing on plates inoculated with suitable dilutions were counted to enumerate each bacterial species present. The threshold of significance was set at ≥10^7^/ml (for one or two species only). Isolates were identified by standard laboratory methods.

### Data analysis

Mono-infection was defined as the presence of only one pathogen as determined by viral and bacterial analysis. Patients for which more than one pathogen was detected were considered to have co-infections.

The outcome was scored as severe if the patient died, or presented a wrong clinical evolution during hospitalisation (complication) or the hospitalization was longer than 10 days (3^rd^ quartile).

Analyses were performed with R software [11]. The nonparametric Wilcoxon test was used to compare means. Qualitative variables were compared with the Fisher exact test. Statistical differences were considered significant for p-values<0.05. Multivariate backward step-by-step logistic regressions were performed to take into account confounders, bias and interactions involving pathogens linked to the dependent variable. All variables with a p-value<0.20 were included in the initial model.

Five age groups were defined for the analysis: [0–5] months, [6–12] months,[13–18] months, [19–24] months, [25–36] months, >36 months.

## Results

From November 2010 to July 2012, 290 children were recruited from the 301 hospitalized patients eligible. Eleven eligible patients refused to participate in the study, and they did not differ from included patients for age, sex or diagnosis on admission.

The baseline demographic and clinical characteristics of the patients are shown in Table 1. The sex ratio (male/female) was 1.3. The mean of age was 1.1 years [95% CI: 1.0 to 1.2], the age range was from 1 day to 5 years, and 250 (86%) patients were 2 years old or younger. The diagnoses on admission (Table S1) were significantly different between the age groups: bronchiolitis was found more frequently among patients under 6 months old (55%, p<0.01); LTRI and pneumonia were more frequent among patients aged over 12 months (respectively, 68% and 85%,p<0.01).

**Table 1:** Patient characteristics and main aetiologies according for mono- and co- infections among patients under 5 years old hospitalised for SARI between November 2010 and July 2012 in Antananarivo, Madagascar.

	Total	Mono-infection	Co-infection	p	adjusted OR
	N = 290 (%)	N = 81 (27.9)	N = 192 (66.2)		[95%CI]
**Sex**	163 (56.2)	52 (33.5)	103 (66.4)	0.11	
Male					
Female	127 (43.8)	29 (24.6)	89 (75.4)		
**Age**	111 (38.3)	27(26.7)	74 (73.3)		**2.7 [1.0–7.0]**
0–5 months					
6–12 months	61 (21.0)	24 (40.7)	35 (59.3)		**1.5 [0.5–4.1]**
13–18 months	34 (11.7)	8 (25.0)	24 (75.0)	0.07	**3.4 [1.0–11.2]**
19–24 months	24 (8.3)	3(13.0)	20 (87.0)		**8.0 [1.7–36.7]**
25–36 months	38 (13.1)	9 (25.0)	6 (75.0)		**3.1 [0.9–9.9]**
>36 months	22 (7.6)	10 (45.5)	12 (54.5)		**–**
**Passive smoking**	113 (39.0)	33 (31.1)	73 (68.9)	0.68	
**Number of rooms in the home**	34 (11.7)	5 (15.6)	27 (84.4)	0.07	
1					
>2	256 (88.3)	76 (31.5)	165 (68.5)		
**Total number of inhabitants in household**	198 (68.3)	52 (28.3)	132 (71.7)		
<5					
6–10	87 (30.0)	28 (33.3)	56 (66.7)	0.67	
>10	5 (1.7)	1 (20)	4 (80)		
**Monthly household income**	85 (36.8)	30 (35.3)	55 (64.7)	0.20	
<182$					
182–455$	78 (78.8)	18 (23.1)	60 (76.9)		
>455$	68 (29.4)	17 (25.0)	51 (75.0)		
**Diagnosis on admission**	145 (50.0)	38 (27.7)	99 (72.3)	0.51	
Bronchiolitis					
Lower respiratory tract infection	60 (20.7)	20 (35.1)	37 (64.9)	0.33	
Pneumonia	44 (16.1)	13 (29.6)	31 (70.5)	0.99	
Respiratory distress	10 (3.5)	2 (22.2)	7 (77.8)	0.97	
**Comorbidity** *	47 (17.2)	10 (21.3)	37 (78.7)	0.22	
**Atopic** **	52 (17.9)	16 (32.0)	34 (68.0)	0.73	
**Hospitalisation before admission**	71 (24.5)	20 (29.0)	49 (71.0)	0.98	
**Antibiotic prior to hospitalisation**	131 (45.2)	43 (35.0)	80 (65.0)	0.09	**0.5 [0.3–0.9]**
**Time to hospitalisation >10 days**	50 (17.2)	13 (30.2)	30 (69.8)	0.97	
**Death**	9 (3.1)	1 (12.5)	7 (87.5)	0.44	
**Pathogens**	130 (44.8)	36 (44.4)	94 (49.0)	0.51	
Respiratory Syncitial Virus					
Influenza A	71 (24.5)	7 (8.6)	64 (33.3)	<0.01	**7.9 [3.2–19.3]**
Rhinovirus	49 (16.9)	7 (8.6)	42 (21.9)	<0.01	**4.4 [1.7–11.2]**
*Streptococcus pneumoniae*	103 (35.5)	7 (8.6)	96 (50.0)	<0.01	**11.1 [4.7–26.6]**
*Haemophilus influenzae de type B*	39 (13.5)	1 (1.2)	38 (19.8)	<0.01	**20.5 [2.6–161.2]**

P value: Fisher's exact test (univariate analysis), OR adjusted: multivariate analysis (variables with p<0.20 on univariate analysis).

–: reference variable.

* ♦Comorbitidy: congenital diseases, malnutrition, prematurity,

** Atopic: including atopic food, drug, acaria pollen or asthma comorbidity.

Forty-nine (17%) patients had at least one underlying disease, with asthma being the most frequent (5%). Some of the children had underlying conditions, particularly atopy (52; 18%) and passive exposure to tobacco smoke (113; 39%). Seventy-one (24%) had been hospitalized during the previous 12 months. Patients aged under 18 months had significantly more prior hospitalisations (p = 0.01). The difference between sexes was not statistically significant.

Around 45% (131/290) of patients had been treated with antibiotics before hospitalisation (Table S2). Significantly more patients under than over 12 months old had received antibiotic treatment before hospitalisation (p<0.01). The difference between sex was not statistically significant. The frequency of bacterial detection was lower in the group with prior antibiotic treatment (49% versus 65%, p = 0.01). No such relationship was found for viral results.

Accurate information about immunisation was available on vaccination cards for 82 (26%) children. Eighty-seven percent of children had completed vaccination against BCG, 77% had received one *Haemophilus influenzae* type B vaccination. No child was vaccinated against *Streptococcus pneumoniae* and only two were vaccinated against influenza.

The average of duration of hospitalisation was 7.9 days [95% CI: 7.1 to 8.6], range 1 to 79 days. The median duration of hospitalisation was 7 days, and 61% of patients were hospitalised for more than 7 days. The duration of hospitalisation for living patients was 8.0 [7.2–8.7] days, and for fatal cases was 3.9 [1.4–9.2]. There were no significant differences between sex and age groups.

Discharge information was available for all cases: 79% (230/290) of patients fully recovered without any complication during their hospital stay; the condition of 60 children worsened during hospitalisation, and nine patients (3%) died. All deaths were of children under 18 months of age, and six (67%) were of children less than 6 months old.

Mono-infection was found in 81 patients (28%), co-infection was found in 192 patients (66%). No infectious agents were found in samples from 17 patients (6%). Overall, 563 pathogens were isolated during the study period (356 viruses and 207 bacteria) (table 2).

**Table 2:** Aetiological agents and co-infections among patients under 5 years old hospitalised for SARI, November 2010 to July 2012 in Antananarivo, Madagascar.

	RSV	IA	RhV	IB	AdV	hMPV	Co	BoV	PiV	*Spn*	*Hib*	*Sta. aur*	*Brc*	*S*	*Acin. bau*	*Aer. hydro*	*Aeroc. spp*	*Pseu. aer*	*Ent. spp*	*List. sp*	*Kleb pn*	*Sta*	*Ser. mar*	*Mora. sp*	*Esch. c*
RSV	**36**	31	12	6	5	2	8	5	1	40	20	3	5	7	1	1	0	0	2	0	3	2	0	1	1
IA	-	**6**	9	9	4	3	2	2	2	28	7	1	3	6	0	0	0	0	0	0	2	1	0	0	0
RhV	-	-	**7**	3	6	2	2	4	2	18	7	0	1	2	0	0	0	1	1	0	2	1	0	1	0
IB	-	-	-	**2**	1	1	1	0	0	5	2	0	0	1	0	0	0	0	0	0	0	1	0	0	0
AdV	-	-	-	-	**4**	0	1	1	0	8	3	0	2	1	0	0	0	0	0	1	0	1	0	0	1
hMPV	-	-	-	-	-	**6**	1	0	2	10	3	1	1	1	0	0	0	0	0	0	0	1	0	0	1
Co	-	-	-	-	-	-	**2**	1	1	7	3	0	1	2	0	0	0	0	1	0	0	0	0	0	0
BoV	-	-	-	-	-	-	-	**0**	0	4	2	1	0	1	0	0	0	0	1	0	0	0	0	0	0
PiV	-	-	-	-	-	-	-	-	**0**	4	3	0	1	2	0	1	0	0	0	0	1	0	0	0	0
*S.pn*	-	-	-	-	-	-	-	-	-	**7**	25	2	3	2	0	0	0	0	0	0	3	1	0	1	0
*Hib*	-	-	-	-	-	-	-	-	-	-	**1**	1	0	4	0	0	0	0	0	0	0	1	0	0	0
*Sta. aur*	-	-	-	-	-	-	-	-	-	-	-	**0**	0	0	0	0	0	0	1	0	0	0	0	0	0
*Brc*	-	-	-	-	-	-	-	-	-	-	-	-	**1**	1	0	0	0	0	0	0	0	0	1	0	0
*S*	-	-	-	-	-	-	-	-	-	-	-	-	-	**5**	0	0	0	0	0	0	0	0	0	0	0
*Acin bau*	-	-	-	-	-	-	-	-	-	-	-	-	-	-	**0**	0	0	0	0	0	0	0	0	0	0
*Aer. hydro*	-	-	-	-	-	-	-	-	-	-	-	-	-	-	-	**0**	0	0	0	0	0	1	0	0	0
*Aeroc. spp*	-	-	-	-	-	-	-	-	-	-	-	-	-	-	-	-	**1**	0	0	0	0	0	0	0	0
*Pseu aer*	-	-	-	-	-	-	-	-	-	-	-	-	-	-	-	-	-	**1**	0	0	0	0	0	0	0
*Ent. spp*	-	-	-	-	-	-	-	-	-	-	-	-	-	-	-	-	-	-	**0**	0	0	1	0	0	0
*List.sp*	-	-	-	-	-	-	-	-	-	-	-	-	-	-	-	-	-	-	-	**0**	0	0	0	0	0
*Kleb pn*	-	-	-	-	-	-	-	-	-	-	-	-	-	-	-	-	-	-	-	-	**0**	0	0	0	0
*Sta*	-	-	-	-	-	-	-	-	-	-	-	-	-	-	-	-	-	-	-	-	-	**0**	0	0	0
*Ser.mar*	-	-	-	-	-	-	-	-	-	-	-	-	-	-	-	-	-	-	-	-	-	-	**0**	0	0
*Mora sp*	-	-	-	-	-	-	-	-	-	-	-	-	-	-	-	-	-	-	-	-	-	-	-	**0**	0
*Esch c*	-	-	-	-	-	-	-	-	-	-	-	-	-	-	-	-	-	-	-	-	-	-	-	-	**0**
Monoinfection	36	6	7	2	4	6	2	0	0	7	1	0	1	5	0	0	1	1	0	0	0	0	0	0	0
2 pathogens	48	34	18	6	10	9	7	3	6	50	10	2	5	4	0	1	0	1	3	1	0	1	1	0	2
3 pathogens	30	17	16	5	4	10	5	4	5	28	17	2	4	7	1	1	0	0	2	0	2	1	0	2	1
4 pathogens	11	12	6	2	3	0	2	1	2	14	7	0	2	3	0	0	0	0	0	0	3	1	0	0	0
5 pathogens	5	2	2	2	2	0	2	2	0	4	4	1	0	1	0	0	0	0	0	0	1	1	0	0	0
**Total**	**130**	**71**	**49**	**17**	**23**	**25**	**18**	**10**	**13**	**103**	**39**	**5**	**12**	**20**	**1**	**2**	**1**	**2**	**5**	**1**	**6**	**4**	**1**	**2**	**3**

*RSV : Respiratory Syncitial Virus; IA : Influenza A; RhV : Rhinovirus; hMPV : human metapneumovirus;Co : coronavirus_co43/NL63/229E; BoV : Bocavirus; PiV : parainfluenza virus 1/2/3; Spn : Streptococcus pneumoniae; Hib : Haemophilus influenzae* de type b; *Sta : Staphylococcus aureus; Brc : Branhamella catharralis; S : Streptococcus mitis/sanguinis/G/D/equinis/Beta haemolitic; Aero.h : Aero hydromonas; Aeroc spp : Aerococcus spp; Pseu aer : Pseudomonas aeruginosa; List sp : Listeria sp; Ent.spp : Enterobacter spp; Kleb pn : Klebsiella pneumoniae; Ser Mar : Serritia marcescens; Mora sp : Moraxela species; Esch. c : Escherichia coli.*

RSV was the most frequently detected virus (37%, 130/356), followed by Influenza type A virus (20%, 71/356) and Rhinovirus (14%, 49/356) (Table 2).

*Streptococcus pneumoniae* was most frequently detected bacterial species (50%, 103/207), followed by *Haemophilus influenzae* type B (19%, 39/207) (table 2).

For the patients who died, *Streptococcus pneumoniae* (56%, 5/9) was the pathogen most frequently detected, followed RSV (33%, 3/9) and Influenza type A virus (22% 2/9).

For multivariate analysis of factors possibly associated with co-infection, sex, age group, number of rooms in the home and antibiotic treatment before hospitalisation were included in the initial model.

The multivariate analysis indicated that children under 36 months of age were more often infected by more than one pathogen; the difference was statistically significant for the 19–24 months old group (adjusted OR = 8.0) (Table 1). Previous antibiotic treatment before hospitalization was a significantly protective factor against co-infection (OR = 0.5).

In univariate analysis, Influenza A, Rhinovirus, *Streptococcus pneumoniae*, and *Haemophilus influenza* type B were significantly associated with co-infections. *Haemophilus influenzae* and *S. pneumoniae* were most often associated with co-infection (Adjusted OR = 20.5 and 11.1, respectively) (Table 1).

Univariate analysis showed that a severe outcome was significantly more frequent for patients with comorbidity (OR = 3.9), including prematurity or congenital disease (such as heart disease or cerebral malformation). Also, two of the three patients presenting with malnutrition died. Hospitalization during the 12 months prior to admission was also associated with a severe outcome (OR = 2.1). A low household income, of less than 455 US$ (less than 182 US$ OR = 4.4 and between 182 and 455 US$ OR = 3.5) was associated with severe outcomes.

For multivariate analysis, age group, monthly household income, admission diagnosis, comorbidities, previous hospitalisation, and infection with influenza virus type A, RSV and rhinovirus were included in the initial model.

Multivariate analysis (Table 3) adjusted for these variables showed that age below 6 months (OR = 5.3), living in a household with low income (OR = 4.1), and the presence of comorbidities (OR = 4.6) were significantly aggravating factors. Infection with an influenza type A virus was a significantly protective factor against a severe outcome (OR = 0.4 [95% CI = 0.2–0.9]).

**Table 3:** Patient characteristics and aetiologies according to outcome among patients under 5 years old hospitalized for SARI, November 2010 to July 2012, in Antananarivo, Madagascar.

	Amelioration	Severe outcome	crude OR *	p- value	adjusted OR#
	N = 230 (%)	N = 60 (%)	[95%CI]		[95%CI]
**Sex**					
Male	129 (79.1)	34 (20.9)	–	0.99	
Female	101 (79.5)	26 (20.5)	0.9 [0.5–1.7]		
**Age**					
0–5 months	79 (71.2)	32 (28.8)	1.8 [0.6–5.8]		**5.3 [1.3–21.7]**
6–12 months	54 (88.5)	7 (11.5)	0.6 [0.2–2.2]		**1.4 [0.3–6.6]**
13–18 months	27 (79.4)	7 (20.6)	1.2 [0.3–4.6]	0.14	**1.8 [0.4–8.0]**
19–24 months	20 (83.3)	4 (16.7)	0.9 [0.2–4.1]		**1.3 [0.2–7.1]**
25–36 months	32 (84.2)	6 (15.8)	0.8 [0.2–3.4]		**0.8 [0.2–3.9]**
>36 months	18 (81.8)	4 (18.2)	–		**–**
**Passive smoking**	89 (78.8)	24 (21.2)	1.1 [0.6–1.9]	0.88	
**Number of rooms in the home**					
1	26 (76.5)	8 (23.5)	1.2 [0.5–2.8]		
>2	204 (79.7)	52 (20.3)	–	0.66	
**Monthly household income**	64 (71.9)	25 (28.1)	4.4 [1.7–11.5]	<0.01	**4.1 [1.5–11.3]**
<182$					
182–455$	64 (76.2)	20 (23.8)	3.5 [1.3–9.4]		**4.3 [1.5–12.3]**
>455$	68 (91.9)	6 (8.1)	–		**–**
**Diagnosis on admission**					
Bronchiolitis	121 (83.5)	24 (16.5)	0.6 [0.3–1.1]	0.11	**0.5 [0.2–0.9]**
Lower respiratory tract infection	47 (78.3)	13 (21.7)	1.1 [0.5–2.1]	0.86	
Pneumonia	34 (72.3)	13 (27.7)	1.6 [0.8–3.3]	0.23	
Respiratory distress	6 (60.0)	4 (40.0)	2.7 [0.7–9.8]	0.22	
**Comorbidity** **	28 (57.1)	21 (42.9)	3.9 [2.0–7.5]	<0.01	**4.6 [2.1–10.3]**
**Atopic** ***	44 (84.6)	8 (15.4)	0.6 [0.3–1.5]	0.35	
**Hospitalisation before admission**	49 (69.0)	22 (31.0)	2.1 [1.1–3.9]	0.02	
**Antibiotic prior to hospitalisation**	107 (81.7)	24 (18.3)	0.8 [0.4–1.4]	0.39	
**Biological diagnoses**					
Viral infections	199 (80.2)	49 (19.8)	0.7 [0.3–1.5]	0.40	
Bacterial infections	131 (78.4)	36 (21.6)	1.1 [0.6–2.0]	0.77	
**Pathogens**					
Respiratory Syncitial Virus	108 (83.1)	22 (16.9)	0.7 [0.4–1.2]	0.19	
Influenza A	62 (87.3)	9 (12.7)	0.5 [0.2–1.0]	0.06	**0.4 [0.2–0.9]**
Rhinovirus	44 (89.8)	5 (10.2)	0.4 [0.1–1.0]	0.05	
*Streptococcus pneumoniae*	84 (81.5)	19 (18.5)	0.8 [0.4–1.5]	0.55	
*Haemophilus influenzae de type B*	31 (79.5)	8 (20.5)	1.0 [0.4–2.3]	0.97	
**Coinfection**	155 (80.7)	37 (19.3)	1.1 [0.5–2.0]	0.98	
**Coinfection types**					
*Viral co-infection*	40 (93.0)	3 (7.0)	0.2 [0.1–0.9]	0.03	
*Bacterial co-infection*	6 (85.7)	1 (14.3)	0.5 [0.1–4.7]	0.62	
*Viral and bacterial co-infection*	109 (76.8)	33 (23.2)	0.9 [0.5–1.8]	0.97	

* Univarite analysis;

# Multivariate analysis (variables with p<0.20 on univariate analysis).

–: reference variable.

** Comorbitidy: congenital diseases, malnutrition, prematurity.

*** Atopic: including atopic food, drug, acaria pollen or asthma comorbidity.

## Discussion

In the paediatric ward of CENHOSOA, most children under 36 months, hospitalised for respiratory infections, were co-infected, and the most frequently encountered pathogens were *H. influenza*, *S. pneumonia*, Influenza A and Rhinoviruses. During hospitalisation, the evolution of respiratory infections was worse for the youngest children and for those presenting comorbidities and living in low-income household.

Over all, viral infections made a large contribution to the burden of infectious respiratory disease. Viral pathogens were found in 85% of the patients and bacterial pathogens in 57%, although for 45% of the patients had been treated with antibiotics before hospitalisation, and this presumably affected the observed prevalence of bacterial infections.

Our findings for viruses were similar to those of other studies. For example, the detection rate for viral pathogens was 72% in a study in Vietnam [12], 50% in a study in Mozambique [13], both focusing on hospitalised children. These results are consistent with those for the paediatric ward in CENHOSOA. Our findings are also consistent with another report from Madagascar indicating that RSV (30.5%) and by Influenza A (22.6%) are the most frequent viral causes of community respiratory infection [8].

Few studies in developing countries have addressed the role of bacterial co-infection in severe respiratory illness. Most focus on the viral aetiology because molecular diagnosis tools are available for these pathogens allowing to identify the infection in 80%–95% of cases [14], [15]. In developing countries, laboratory methods to identify bacterial pathogens are more difficult to implement, because of the lack of appropriate facilities and material in hospital laboratories and then, long transit times are prejudicial for stability of specimens. In our study the transit time was reduced because of the proximity of the CENHOSOA and the IPM. Unfortunately, our findings for the rate of bacterial infection may be artificially low due to prior antibiotic use. In our study, *Streptococcus pneumoniae* was the most common bacterial pathogen as in other studies of hospitalised patients with acute respiratory illness, and *Haemophilus influenza*e type B was the next most frequent [16]–[19].

Primary infection by a respiratory virus increases the risk of secondary bacterial pneumonia, and viral and bacterial co-infection is common in young children with pneumonia in developing countries (about 20–30% of episodes) [20], [21]. Evidence of probable mixed viral-bacterial infection has been recorded in up to 45% of cases of community acquired pneumonia in children [22]–[25]. A large proportion of our patients had co-infection (70%; 192/273) and the [0–6] months age group had the highest risk. These results are consistent with a study of patients with community respiratory infection in Madagascar [8]. Viral and bacterial co-infection was found in 57% of our patients; the most frequent combination was *Streptococcus pneumoniae* with one of several respiratory viruses (88%). Data from Gambia and Nigeria indicate that mixed bacterial and viral infections may occur in 8 – 40% of cases of childhood pneumonia [26], [27]. Recent data from South Africa indicate that in at least 31% of cases, viral-associated pneumonia may be due to concurrent infection with *Streptococcus pneumoniae* in the absence of vaccination with pneumococcal conjugate vaccines [28].

Appropriate management of respiratory infections can have a significant impact on child mortality and it is important to target priority groups of children most at risk of dying: these groups include very young infants, children infected with HIV and malnourished children [29]. In our study, the outcome was severe for all of the children who presented malnutrition and 67% (2/3) died. Chisti et al show that the combination of pneumonia with severe or moderate malnutrition is associated with a high risk of death: the RR ranges from 2.9 to 121.2 for severe malnutrition, and from 2.5 to 15.1 for moderate malnutrition. For moderate malnutrition, RR = 1.2 to 36.5. Several other studies also lead to the same results [30]–[33]. Ballard et al. report that malnutrition and level of parental education both have an impact on the incidence of respiratory infections [34].

Pneumonia is the leading causes of childhood mortality, and is responsible for approximately 21% of deaths of African children under five years of age each year. However, little information is available about the causes of childhood pneumonia in developing countries. In our study, only 3% of the patients died; this value is low. Note that the study population consists of patients from families with a socioeconomic level that is above the average for the general population in Madagascar. The patients who died were more frequently co-infected and under 6 months old. These results are consistent with other studies on this topic in developing countries [35], [36]. However, study design may have an impact on the mortality rate observed: indeed all fees for hospitalisation were paid by the study to allow follow-up to be optimised.

The study identified living in a low-income household as a risk factor for severe outcome. This is important for patient management during hospitalisation and also highlights the consequences of financial barriers to access to vaccination against influenza and pneumococci. These vaccinations were not included in the panel of vaccinations in expanded programme of immunisation (EPI) during this period in Madagascar. They were only available in private centres and were expensive, and therefore inaccessible for much of the population. Only two of the patients included in this study had received vaccination against influenza and none had received vaccination against pneumococci according to their vaccination cards.

Our statistical analysis identified influenza A infection as a protective factor against a fatal outcome; however, it is well-known that influenza A may have an impact on high mortality during major epidemics [37]–[39].

The co-circulation of influenza A virus, RSV and the high frequency of *Streptococcus pneumoniae* in co-infections in children aged less than 5 years raised issues about the indirect impact of influenza vaccination on respiratory infections. In particular, the benefits of establishing a system of vaccination against infectious agents for which a vaccine is available should be considered to provide access to a larger proportion of the population.

In conclusion, we advocate to further research in developing countries to document in more detail the impact of viral and bacterial co-infections on the prognosis of severe respiratory illness. As numerous infections found in our study could be avoided by immunisation, we recommend that appropriate vaccination must be rapidly available for all the Malagasy children. This would reduce mortality among the youngest children, as this age group currently suffers the greatest burden of morbidity and mortality associated with respiratory infections.

## Supporting Information

Table S1 **Diagnosis at admission according with age groups.**
(DOCX)

Table S2 **Risk factors according with antibiotic treatments before hospitalization.**
(DOCX)
