# Supplementary material for: Outcome Risk Factors during Respiratory Infections in a Paediatric Ward in Antananarivo, Madagascar 2010–2012
Source: PLoS One. 2013 Sep 12;8(9):e72839. doi: 10.1371/journal.pone.0072839 (PMC3771918; doi:10.1371/journal.pone.0072839)
Supplement: Table S1 — Diagnosis at admission according with age groups. (DOCX) [file pone.0072839.s001.docx]

Supplemental file 1. Diagnosis at admission according with age groups.

|  |  | Bronchiolitis | | Pneumonia | | LTRI | |
| --- | --- | --- | --- | --- | --- | --- | --- |
|  | N=290 | n (%) | P value | n(%) | P value | n(%) | P value |
| **Age groups** 0-5 months | 111 | 80 (55) | <0.01 | 1 (7.5) | <0.01 | 7 (12) | <0.01 |
| 6-12 months | 61 | 37 (26) |  | 1 (7.5) |  | 12 (20) |  |
| >12 months | 118 | 28 (19) |  | 11 (85.0) |  | 41 (68) |  |
