# Supplementary material for: Outcome Risk Factors during Respiratory Infections in a Paediatric Ward in Antananarivo, Madagascar 2010–2012
Source: PLoS One. 2013 Sep 12;8(9):e72839. doi: 10.1371/journal.pone.0072839 (PMC3771918; doi:10.1371/journal.pone.0072839)
Supplement: Table S2 — Risk factors according with antibiotic treatments before hospitalization. (DOCX) [file pone.0072839.s002.docx]

|  | Total | | Antibiotic prior hospitalization | | | | | p | |
| --- | --- | --- | --- | --- | --- | --- | --- | --- | --- |
|  | N=290 (%) | | Yes  N=131(45.2) | | No  N=159 (54.8) | | |  | |
| **Sex** | | | | | | | | | |
| Male | 163 | (56.2) | 72 | (55.0) | 91 | (57.2) | 0.72 | |  |
| Female | 127 | (43.8) | 59 | (45.0) | 68 | (42.8) |  | |  |
| **Age** |  | |  |  |  |  |  | |  |
| 0-5 months | 111 | (38.3) | 40 | (30.5) | 71 | (44.7) |  |  |  |
| 6-12 months | 61 | (21.0) | 30 | (22.9) | 31 | (19.5) |  | |  |
| 13-18 months | 34 | (11.7) | 21 | (16.0) | 13 | (8.2) | 0.01 | |  |
| 19-24 months | 24 | (8.3) | 17 | (13.0) | 7 | (4.4) |  | |  |
| 25-36 months | 38 | (13.1) | 19 | (14.5) | 19 | (11.9) |  | |  |
| >36 months | 22 | (7.6) | 4 | (3.1) | 18 | (11.3) |  | |  |
| **Passive smoking** | 113 | (39.0) | 53 | (40.5) | 60 | (37.7) | 0.72 | |  |
| **Number of rooms in the home** |  | |  |  |  |  | 0.36 | |  |
| 1 | 34 | (11.7) | 18 | (13.7) | 16 | (10.1) |  |  |  |
| >2 | 256 | (88.3) | 113 | (86.3) | 143 | (89.9) |  |  |  |
| **Total number of inhabitants in household** |  | |  |  |  |  |  | |  |
| <5 | 198 | (68.3) | 90 | (68.7) | 108 | (67.9) |  |  |  |
| 6-10 | 87 | (30.0) | 40 | (30.5) | 47 | (29.6) | 0.59 | |  |
| >10 | 5 | (1.7) | 1 | (0.8) | 4 | (2.5) |  | |  |
| **Monthly household income** |  | |  |  |  |  | 0.72 | |  |
| <182$ | 89 | (36.0) | 37 | (28.2) | 52 | (32.7) |  |  |  |
| 182-455$ | 74 | (30.0) | 35 | (26.7) | 39 | (24.5) |  |  |  |
| >455$ | 84 | (34.0) | 39 | (29.8) | 45 | (28.3) |  |  |  |
| **Diagnosis on admission** |  | |  |  |  |  |  | |  |
| Bronchiolitis | 145 | (50.0) | 59 | (45.0) | 86 | (54.1) | 0.16 | |  |
| Lower respiratory tract infection | 60 | (20.7) | 34 | (26.0) | 26 | (16.4) | 0.06 | |  |
| Pneumonia | 47 | (16.2) | 24 | (18.3) | 23 | (14.5) | 0.43 | |  |
| Respiratory distress | 10 | (3.5) | 3 | (2.3) | 7 | (4.4) | 0.52 | |  |
| **Comorbidity** | 49 | (16.9) | 15 | (11.5) | 34 | (21.4) | 0.03 | |  |
| **Atopic** | 52 | (17.9) | 20 | (15.3) | 32 | (20.1) | 0.36 | |  |
| **Hospitalisation before admission** | 71 | (24.5) | 35 | (26.7) | 36 | (22.6) | 0.49 | |  |
| **Time to hospitalisation >10 days** | 50 | (17.2) | 21 | (16.0) | 29 | (18.2) | 0.64 | |  |
| **Death** | 9 | (3.1) | 1 | (0.8) | 8 | (5.0) | 0.04 | |  |
| **Pathogens** |  | |  |  |  |  |  | |  |
| Respiratory Syncitial Virus | 130 | (44.8) | 65 | (49.6) | 65 | (40.9) | 0.16 | |  |
| Influenza A | 71 | (24.5) | 30 | (22.9) | 41 | (25.8) | 0.59 | |  |
| Rhinovirus | 49 | (16.9) | 14 | (10.7) | 35 | (22.0) | 0.01 | |  |
| *Streptococcus pneumoniae* | 103 | (35.5) | 40 | (30.5) | 63 | (39.6) | 0.11 | |  |
| *Haemophilus influenzae de type B* | 39 | (13.5) | 15 | (11.5) | 24 | (15.1) | 0.39 | |  |

Supplemental file 2 Risk factors according with antibiotic treatments before hospitalization
